# Supplementary figures and images for: Functional and Compositional Changes in the Fecal Microbiome of a Shorebird during Migratory Stopover
Source: mSystems. 2023 Feb 14;8(2):e01128-22. doi: 10.1128/msystems.01128-22 (PMC10134852; doi:10.1128/msystems.01128-22)

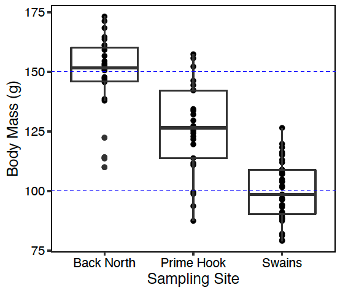

Supplement: FIG S1 [file msystems.01128-22-s0001.tif]

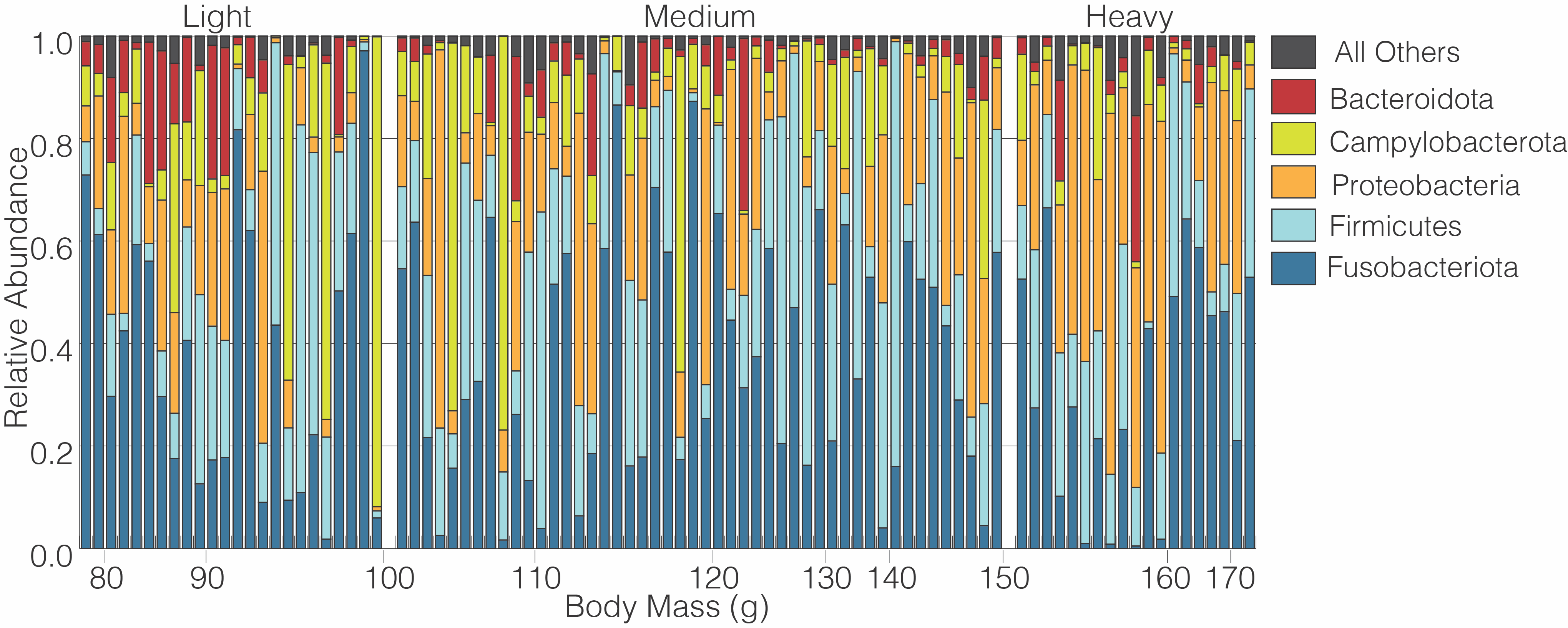

Supplement: FIG S2 [file msystems.01128-22-s0002.tif]
